# Chinese herbal medicine (Tangshen Formula) formula treatment of patients with diabetic kidney disease: a systematic review and meta-analysis

**DOI:** 10.3389/fendo.2025.1522759

**Published:** 2025-06-16

**Authors:** Peng Zhao, Yang Li, Yu Sun, Shiwen Yan, Xiaqing Su, Yunying Sun, Jiacheng Shi, Xiaoping Zhu

**Affiliations:** Haining People’s Hospital, Jiaxing, Zhejiang, China

**Keywords:** diabetic kidney disease, TCM compound, systematic review, meta-analysis, renal function

## Abstract

**Background/Objective:**

Diabetic Kidney Disease (DKD) is a severe complication of diabetes mellitus and is one of the main causes of end-stage renal disease globally. Tangshen Formula (TSF) plays an important role in the treatment of DKD. The purpose of this study was to evaluate the efficacy and safety of TSF compound therapy in treating DKD patients with macroalbuminuria through systematic review and meta-analysis methods.

**Methods:**

Multiple databases, including PubMed, Embase, Cochrane Library and Web of Science, were searched to find eligible RCTs. The main outcome indicators included renal Primary Outcomes(UAER, 24h UP), Secondary Outcomes(eGFR and TCM system scores) and adverse events. Statistical analysis was conducted using RevMan 5.3 software, and the fixed-effect model or random-effect model was selected based on the heterogeneity among the studies.

**Results:**

From 637 retrieved articles, 4 RCTs were finally included, involving 639 participants. The methodological quality of the included studies was generally good. The results indicate that, compared to the sole conventional placebo treatment, the use of TSF treatment after 24 weeks shows significant improvement in the experimental group over the control group, with UAER (MD=-15.94(95% CI: -30.67—1.22); P=0.03) and 24h UP (MD=-0.20(95% CI:-0.36—0.05);P=0.01); assessment of eGFR and scores showed no significant changes in the levels of these two indicators in patients, e GFR (MD=-4.95(95% CI: -11.52–1.62); P=0.47) and scores (MD=0.35(95% CI: -1.29–1.98);P=0.92). Microalbuminuria TSF group and placebo group UAER baselines were similar, with no statistical significance (OR= -4.32, 95% CI (-14.10, 5.48), P=0.29). Macroalbuminuria TSF group and placebo group UAER baselines were similar, with no statistical significance (OR =6.51, 95% CI (-6.27, 19.27), P=0.17). In the TMC compound therapy for DKD patients with massive proteinuria, the results show that the probability of adverse experiments in the intervention group was significantly lower than that in the control group (OR= 0.55 95% CI 0.30-1.03), P=0.79). There was no significant difference between the two groups.

**Conclusion:**

In summary, the findings of this meta-analysis suggest that TSF can provide effective assistance in reducing urinary protein and improving eGFR in DKD patients compared to conventional treatment. These benefits are consistently observed across both microalbuminuric and macroalbuminuric patient cohorts. Due to the limitations in the number and quality of the included studies, the preliminary findings necessitate further validation through high-quality, randomized controlled trials with larger sample sizes and longer follow-up periods to robustly confirm the efficacy of TSF and elucidate its precise mechanisms of action in DKD management.

## 1 Introduction

Diabetic kidney disease (DKD) is a common complication of diabetes mellitus (DM). It is characterized by proteinuria and loss of kidney function (1). In 2021, it was estimated that 536.6 million people worldwide suffered from diabetes, with projections to increase to 783.2 million by 2045, including over 140 million in China alone (2). Diabetic kidney disease is one of the common microvascular complications of diabetes (3), accounting for approximately 30% to 40% of diabetic patients (4). With the continuous rise in the global incidence of diabetes, the prevalence of DKD has also increased (5, 6), posing a significant challenge to individual health and public health. The early symptoms of DKD are not obvious, leading to frequent delays in seeking medical attention or neglecting the importance of screening and regular follow-up, which may ultimately lead to end-stage renal disease or even death (7, 8). Currently, the treatment focus for DKD is on controlling blood sugar and blood pressure, as well as using drugs such as renin-angiotensin system (RAS) blockers to reduce proteinuria and slow the deterioration of kidney function (9). However, the main side effects of ACEIs and ARBs, such as dry cough, hyperkalemia, and elevated serum creatinine, limit their application, especially in patients with a glomerular filtration rate (GFR) <60ml/min/1.73 m^2. As a result, research is increasingly turning to the application of traditional Chinese medicine (TCM) in the treatment of DKD.

The TCM approach to treating DKD is flexible and diverse, emphasizing personalized treatment and holistic regulation. TCM views DKD as a consequence of long-term consumption of sweet fluids (i.e., diabetes), leading to weakness in kidney energy, obstruction due to blood stasis, and internal dampness. Treatment strategies include differential diagnosis and treatment, phased treatment, and the use of classic and empirical formulas, aiming to adjust the body’s yin-yang balance, invigorate the blood and dispel stasis, clear heat and promote diuresis, and nourish the liver and kidneys (10, 11). TSF (Tangshen Formula) is a TCM formula that has received much attention in the research of DKD treatment, composed of various Chinese medicinal materials such as Salvia miltiorrhiza, Astragalus membranaceus, Ligusticum chuanxiong, Angelica sinensis, Rehmannia glutinosa, Dioscorea opposita, Poria cocos, Paeonia rubra, Euryale ferox, and Lycium barbarum. The synergistic effect of these herbs endows TSF with multifaceted pharmacological actions, including anti-inflammatory, antioxidant, anti-fibrotic, reducing proteinuria, and improving kidney function. For example, the anti-inflammatory and antioxidant properties of Salvia miltiorrhiza and Astragalus membranaceus can alleviate damage to the glomeruli and tubular cells while inhibiting the transdifferentiation of tubular epithelial cells, reducing the production of extracellular matrix, and thus alleviating tubulointerstitial fibrosis (12). Additionally, TSF has been demonstrated to enhance the glomerular filtration barrier function, thereby reducing proteinuria and promoting the tubular reabsorption of sodium and water. This multifaceted mechanism further contributes to the overall improvement of renal function. These findings collectively highlight the therapeutic potential of TSF in the management of diabetic kidney disease (DKD) and provide robust scientific evidence supporting the application of TCM in this clinical context. Lu et al. conducted a cohort study involving 150 patients with DKD, who were treated with Ye’s Tangshen Formula in combination with umbilical therapy. The findings demonstrated that Ye’s Tangshen Formula effectively alleviated clinical symptoms, reduced 24-hour urinary protein excretion, and delayed the progression of renal failure in patients with clinically staged diabetic nephropathy characterized by spleen-kidney qi deficiency, dampness, turbidity, and blood stasis. Notably, the incorporation of umbilical therapy significantly decreased the Traditional Chinese Medicine (TCM) syndrome score (13).

Despite related research, the current treatment outcomes for DKD remain unsatisfactory, underscoring the necessity to explore alternative treatment options. In this study, we conducted a meta-analysis to assess the efficacy and safety of TSF compared to placebo in treating patients with DKD. thereby providing evidence-based insights to guide clinical practice and future research.

## 2 Materials and methods

### 2.1 Database and search strategies

#### 2.1.1 Inclusion and exclusion criteria

Studies were selected for inclusion by two independent reviewers, and were approved by a third reviewer. Inclusion criteria (1): RCTs design (2); DKD patients without restriction on age or medical conditions (3); intervention using probiotics (4); assessment of renal function injury, glucose and lipid metabolism, inflammation and oxidative stress mediator concentrations as an outcome variable. Exclusion criteria (1): Non-RCTs design, such as observational studies, reviews, meta-analyses, short reports, conference papers, research projects, or animal trials (2). RCTs with improper statistical methods, incomplete data, and undescribed data with mean and standard deviation (3); Literature of poor quality or without full text.

#### 2.1.2 Data extraction

After meeting the inclusion and exclusion criteria, the included studies were independently reviewed by two reviewers using a standardized template independently. The subsequent data were abstracted which including but not limited to (1): basic features: author, year, country, age, target population, intervention, study duration, and outcome biomarkers (2); methods: randomization, allocation concealment, blindness, data integrity, selective reporting, and other biases (3); research objects: patients with DKD were divided into an experimental group and a control group (4); intervention measures: specific medication, dose, treatment duration (5); outcome biomarkers: renal function: serum creatinine (Scr), BUN, GFR, 24-h urine protein (24 h-UP), urinary albumin/creatinine ratio (UACR), cystatin C (Cys C), potassium (K), natrium (Na); glucose metabolism: FPG, 2 h postprandial blood glucose (2 h-PBG), insulin, HbA1c, HOMA-IR, QUICKI; lipid metabolism: TG, TC, low-density lipoprotein cholesterol (LDL-c), VLDL-c, HDL-c; inflammation and oxidative stress: serum high-sensitivity C-reactive protein (hs-CRP), plasma malondialdehyde (MDA), total antioxidant capacity (TAC), GSH and NO.

#### 2.1.3 Search strategy

A literature search was conducted using eight electronic databases, including the Web of Science, PubMed, Cochrane Library, Embase, Chinese Biological Medicine Database (CBM), China National Knowledge Infrastructure (CNKI), Chinese Scientific Journal Database (VIP) and the Wanfang database, for original articles published before February 30, 2024. The search terms included: ((Tangshen Formula) AND (diabetic kidney disease OR Nephropathies, Diabetic OR Diabetic Kidney Disease OR Kidney Diseases, Diabetic OR Intracapillary Glomerulosclerosis OR Kimmelstiel Wilson Syndrome OR Kimmelstiel-Wilson Disease(MeSH Terms))) OR ((Chinese herbal medicine) AND (diabetic kidney disease OR Nephropathies, Diabetic OR Diabetic Kidney Disease OR Kidney Diseases, Diabetic OR Intracapillary Glomerulosclerosis OR Kimmelstiel Wilson Syndrome OR Kimmelstiel-Wilson Disease(MeSH Terms))).

Studies involving patients with mixed malignancies, non-controlled trials, non-clinical studies, literature reviews, meta-analyses, meeting abstracts, case reports, duplicate studies, experimental models and those with insufficient available data were excluded.

#### 2.1.4 Quality assessment

The researchers evaluated the quality of the literature independently. The Cochrane bias risk assessment tool was used to assess methodological quality. Evaluation aspects included whether (1): random sequences were properly generated (2); the distribution of hidden was properly used (3); subjects and intervention providers were properly blinded (4); evaluators of the results were properly blinded (5); the completeness of outcome data was properly maintained (6); selective reporting was properly conducted (7); other biases were properly disposed. According to the above specific evaluation criteria, the included studies were categorized as ‘low risk’, ‘high risk’ or ‘unclear risk’.

#### 2.1.5 Statistical analysis

RevMan 5.3 were used for statistical analysis and graph of risk of bias. The effect of probiotics on selected parameters was analyzed using the mean difference with standard deviations (SDs). The weighted mean difference (WMD) was adopted when the same measurement unit or method was applied for the same intervention. Otherwise, the standardized mean difference (SMD) was employed. When the study’s authors did not provide SDs of mean differences, SDs for the changes from baseline were substituted using a correlation coefficient calculated according to Cochrane recommendations and the SDs for the baseline and final means for each group. Therefore, we calculated the SDs of outcomes using the following formula: SD2 change = SD2 baseline + SD2 final – (2 × correlation coefficient × SD baseline × SD final), assuming that the correlation coefficient I was 0.5 (14). Interval estimation adopted a 95% confidence interval (CI). I^2^ ≤ 50% and p ≥ 0.05 implied a lack of heterogeneity; therefore, the fixed-effect model was used to combine the effect value. I^2^ > 50% and p < 0.05 indicated the existence of heterogeneity; hence, the random-effect model was used. Furthermore, potential publication bias in each analysis was assessed quantitatively using funnel plots (15). If publication bias existed, the results were reported truthfully after considering the sensitivity analysis results (16). Sensitivity analysis was performed based on the characteristics of the study. A p value of 0.05 was considered as level of statistical significance.

Review Manager (RevMan) version 5.3 (Nordic Cochran Centre, Copenhagen, Denmark) was used for statistical analyses. Data were mainly expressed as odds ratio (OR) with corresponding 95% confidence interval (CI), and a two-tailed P<0.05 was considered to be statistically significant. Cochrane’s Q test and I^2^ statistics were used to assess heterogeneity among the studies: if P> 0.1 or I^2^ <50%, fixed-effects model was used for the meta-analysis; otherwise, random-effects model was used (16). The presence of publication bias was investigated using the funnel plots. A pooled analysis of publication bias determined that the trim-and-fill method should be applied to coordinate the estimates from unpublished studies, and the adjusted results were compared with the original pooled OR (17, 18). Sensitivity analysis was performed to evaluate the impact of different therapeutic regimens, sample size, and type of research on the clinical efficacy of the combination of conventional treatment and JLC.

## 3 Results

### 3.1 Study search results and study characteristics

**Figure 1:**
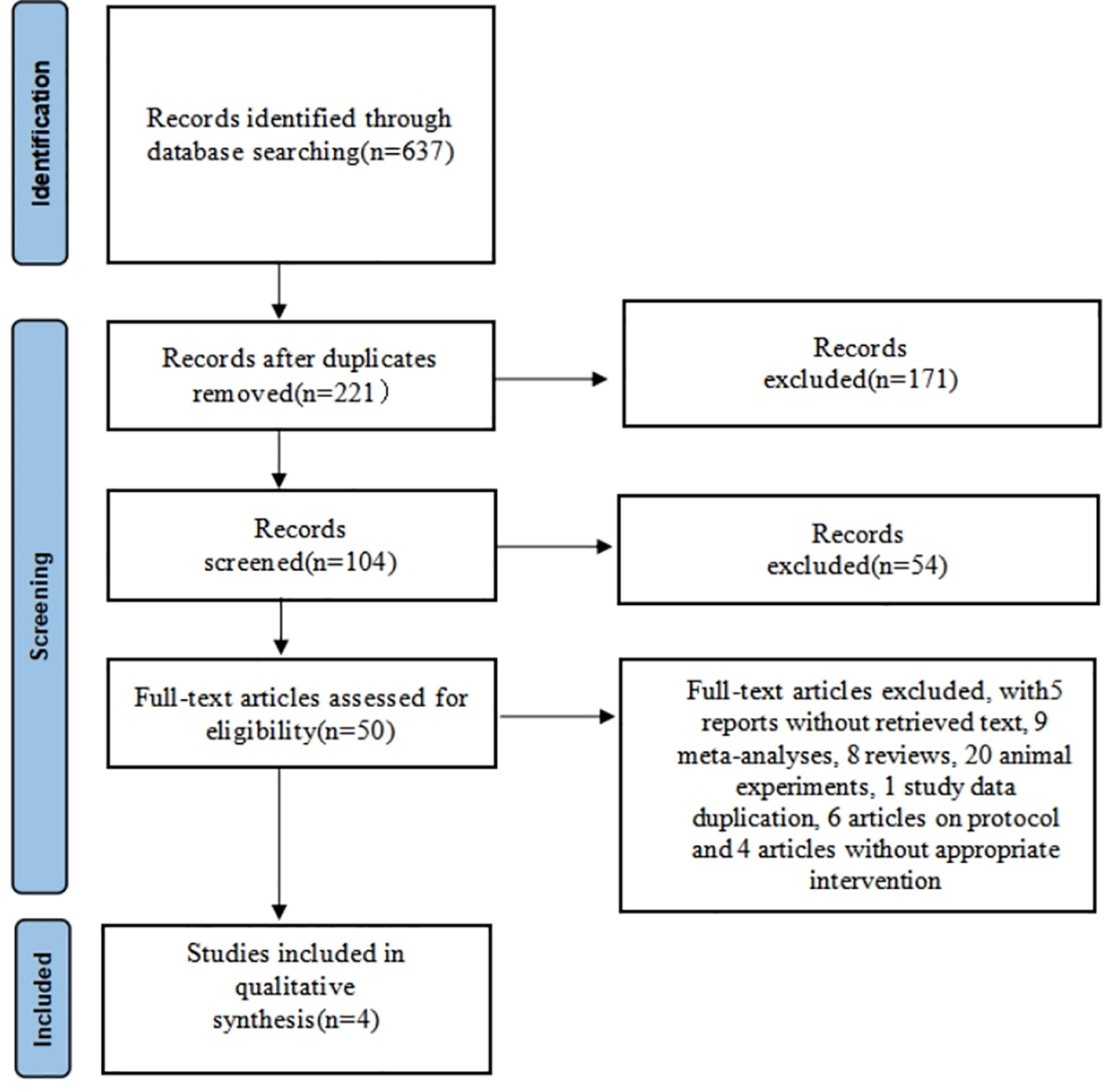
PRISMA flow diagram of the study selection process.

**Figure 2:**
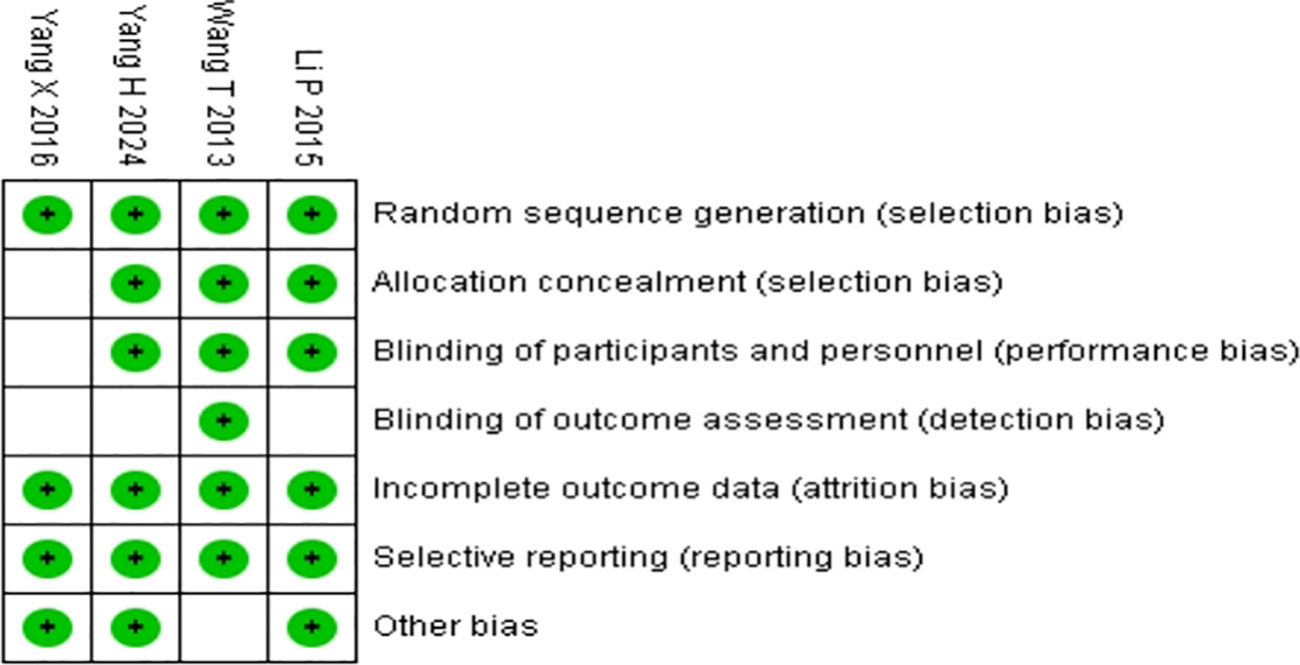
Quality assessments of the included RCTs articles:risk of bias summary for all RCT studies. Each color represents a different level of bias: red for high-risk, green for low-risk, and white for unclear-risk of bias.

A total of 637 articles were searched through literature retrieval. After carefully reviewing the titles, abstracts, duplications and relevance, we retained 50 articles for further review. Interrater agreement in determining the final studies from the 50 screened citations was substantial (κ = 0.637). 5 reports without retrieved text, 9 meta-analyses, 8 reviews, 20 animal experiments, 1 study data duplication, 6 articles on protocol and 4 articles without appropriate intervention were further excluded. In the end, 4 RCTs were included for meta-analysis, containing 9 English articles and 1 Chinese article. The 4 RCTs incorporated a total of 639 participants (intervention, 383; control, 256) (**Figure 1**). The characteristics of all included RCTs were summarized in **Tables 1**, **2**, with their methodological quality highlighted in **Figure 2**.

**Table 1:** Basic features of included studies.

Location	Number	Therapy condition	Condition type	Number experiment/Control	Age	Research type	Literature rating	Reference
Wang T 2013	60	Chinese herbal medicine Tangshen Formula	last 24 weeks	30/30	18–65 years	Controlled trial	8	(19)
Yang H 2024	219	Tangshen Formula	last 24 weeks	109/110	49–54 years	Controlled trial	7	(20)
Yang X 2016	180	Tangshen Formula	last 24 weeks	122/58	25–75 years	Controlled trial	8	(21)
Li P 2015	180	Tangshen Formula	last 24 weeks	122/58	25–75 years	Controlled trial	7	(22)

**Table 2:** Clinical information from the eligible trials in the meta-analysis.

Included studies	Dosage of TSF	Intervention period	Intervening methods	Control group regimens	Parameter types	Reference
Wang T 2013	8 grams TSF, twice daily	24 weeks	TSF VS Con	placebo	glycosylated hemoglobin (HbA1c),24-hour total urine protein (24-hTP)	(19)
Yang H 2024	8 grams TSF, twice daily	24 weeks	TSF VS Con	placebo	hemoglobin A1c (HbA1c), albumin-to-creatinine ratio (UACR) levels,8-hydroxy-2′-deoxyguanosine (8-OHdG), 3-nitrotyrosine (3-NT), the enzymic anti-oxidant, superoxide dismutase (SOD)	(20)
Yang X 2016	8 grams TSF, twice daily	24 weeks	TSF VS Con	placebo	Scr (μmol/L),24 h UP (g/24 h),eGFR (ml/min/1.73 m2), In plasma L-FABP (μg/ml)	(21)
Li P 2015	8 grams TSF, twice daily	24 weeks	TSF VS Con	placebo	ORR,AE,UAER (μg/min),Scr (μmol/L),24 h UP (g/24 h),eGFR (ml/min/1.73 m2),BUN (mmol/L)	(22)

Scr (μmol/L), Serum Creatinine; 24 h UP (g/24 h), 24-Hour Urine Protein; eGFR, Estimated Glomerular Filtration Rate; ORR, Objective Response Rate; AE, Adverse Event; UAER, Urine Albumin Excretion Rate; BUN, Blood Urea Nitrogen; L-FABP, Liver Fatty Acid Binding Protein.

Meta-analyses of Chinese herbal medicine Formula treatment with diabetic kidney disease.

Analysis **Table 1**, all included studies were conducted in different medical centers in China. The intervention group consisted of 363 patients treated with TSF Formula. The control group had 256 patients who were routinely treated with a placebo. The quality standards of the drugs used in this study have been approved by the State Food and Drug Administration (SFDA) of China, and have been granted a manufacturing license number issued by the Chinese SFDA (Z10980041). All involved pharmaceutical companies followed the quality processing procedures outlined in the pharmacopoeia. The study and patient characteristics are summarized in **Table 1**.

### 3.2 Quality of included studies

Quality assessment of the risk of bias is shown in **Supplementary Figure 2** and **Table 3**. The results revealed that the literature retrieved for the present study was of good quality.

**Table 3:** The level of proof synthesis.

Location	Study type	Random sequence generation	Allocation concealment	Blinding of participants and researchers	Blinding of outcome assessment	Incomplete outcome data	Selective reporting	Other bias	Eligibility for review	Overall level of proof
Wang T 2013	Controlled trial	√	√	√	√	√	√	?	Yes	High
Yang H 2024	Controlled trial	√	√	√	?	√	√	√	Yes	High
Yang X 2016	Controlled trial	√	?	?	?	√	√	√	Yes	Intermediate
Li P 2015	Controlled trial	√	√	√	?	√	√	√	Yes	High

x, high risk of bias; ?, insufficient information; √, low risk of bias.

Analyzing **Figure 2** and **Table 3**, the study by Wang T in 2013 is unbiased on all listed criteria, with an overall high level of evidence. The study by Yang H in 2024 has uncertainties in “Blinding of outcome assessment,” but is unbiased on other criteria, with an overall high level of evidence. The study by Yang X in 2016 has uncertainties in “Allocation concealment,” “Blinding of participants and researchers,” and “Blinding of outcome assessment,” with an overall moderate level of evidence. The study by Li P in 2015 has uncertainties in “Blinding of outcome assessment,” but is unbiased on other criteria, with an overall high level of evidence.

### 3.3 Quantitative data analysis

Analyzing **Table 2**, all studies adopted the same TSF dosage: 8 grams, twice daily (19–22). The intervention period for all studies was 24 weeks (19–22). The intervention method in all studies involved comparing TSF with conventional treatment (Con) (19–22), while the control group received a placebo. The assessment parameters employed in the studies were heterogeneous, encompassing a range of biomarkers associated with diabetes and kidney function. This diversity in biomarker selection underscores the complexity in evaluating the efficacy and safety profile of TSF. Through comparative analysis of the TSF group versus the placebo group, researchers were able to systematically assess the therapeutic impact of TSF on diabetes and its associated nephropathies.

#### 3.3.1 Primary outcomes assessment

**Figure 3:**
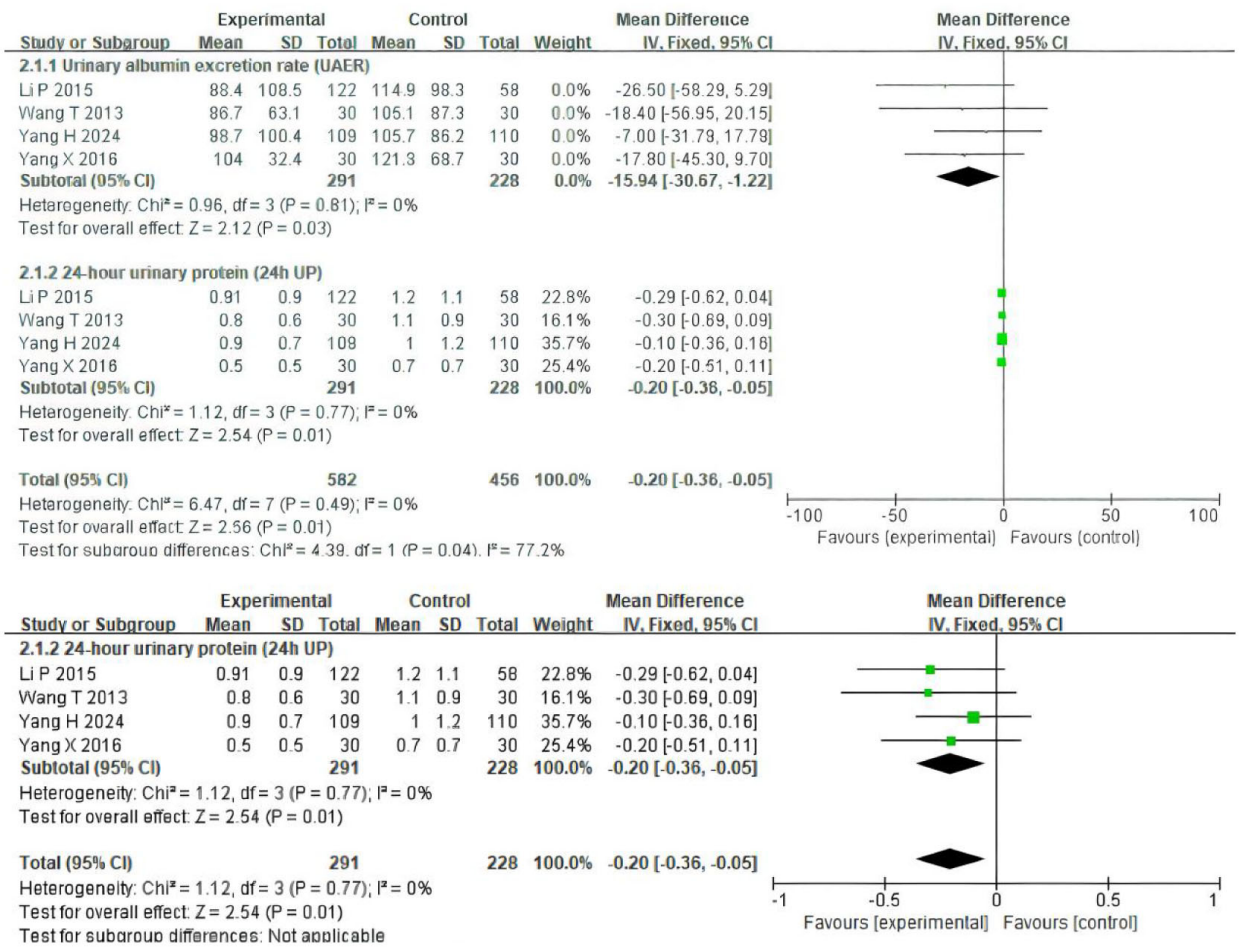
Comparisons of the values of UAER and 24 UP between experimental and control group (after 24 weeks).

Four trials (19–22) involving 519 participants evaluated UAER and 24h UP data (**Figure 3**). The experimental group was treated with TSF, while the control group received placebo treatment as usual. After 24 weeks of treatment, the results showed significant improvement in the experimental group compared to the control group, with UAER (MD=-15.94(95% CI -30.67 -1.22); P=0.03) and 24h UP (MD=-0.20(95% CI -0.36 -0.05); P=0.01). There was no heterogeneity between studies for UAER (P=0.81, I^2^ = 0%) and 24h UP (P=0.77, I^2^ = 0%), so a fixed-effect model was used to analyze OR; otherwise, a random-effects model was used.

#### 3.3.2 Secondary outcomes assessment

**Figure 4:**
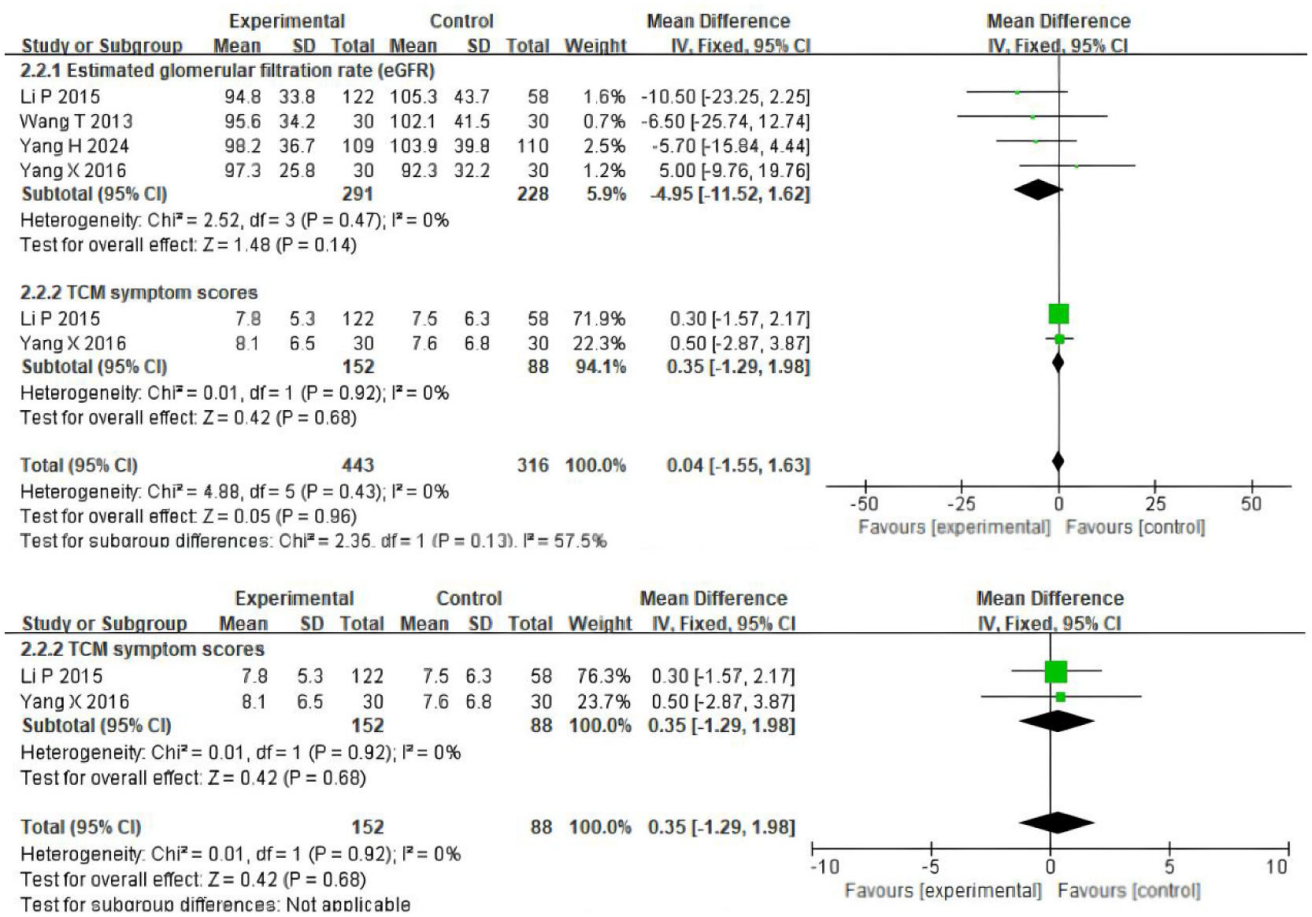
Comparisons of the values of eGFR and system scores between experimental and control group (after 24 weeks).

Four trials (19–22) involving 519 participants evaluated eGFR, and two trials (21, 22) included 240 patients reporting scores data (**Figure 4**). Patients in the experimental group were treated with TSF, while those in the control group were conventionally treated with placebo. After 24 weeks of treatment, the results showed no significant changes in the levels of two outcome indicators in the patients’ bodies: eGFR (MD=-4.95(95% CI -11.52–1.62); P=0.47) and scores (MD=0.35(95% CI -1.29–1.98); P=0.92). There was no heterogeneity between studies for eGFR (P=0.47, I^2^ = 0%) and scores (P=0.92, I^2^ = 0%), so a fixed-effect model was used to analyze OR; otherwise, a random-effects model was used.

### 3.4 The assessment of eGFR outcomes (microalbuminuria, macroalbuminuria)

**Figure 5:**
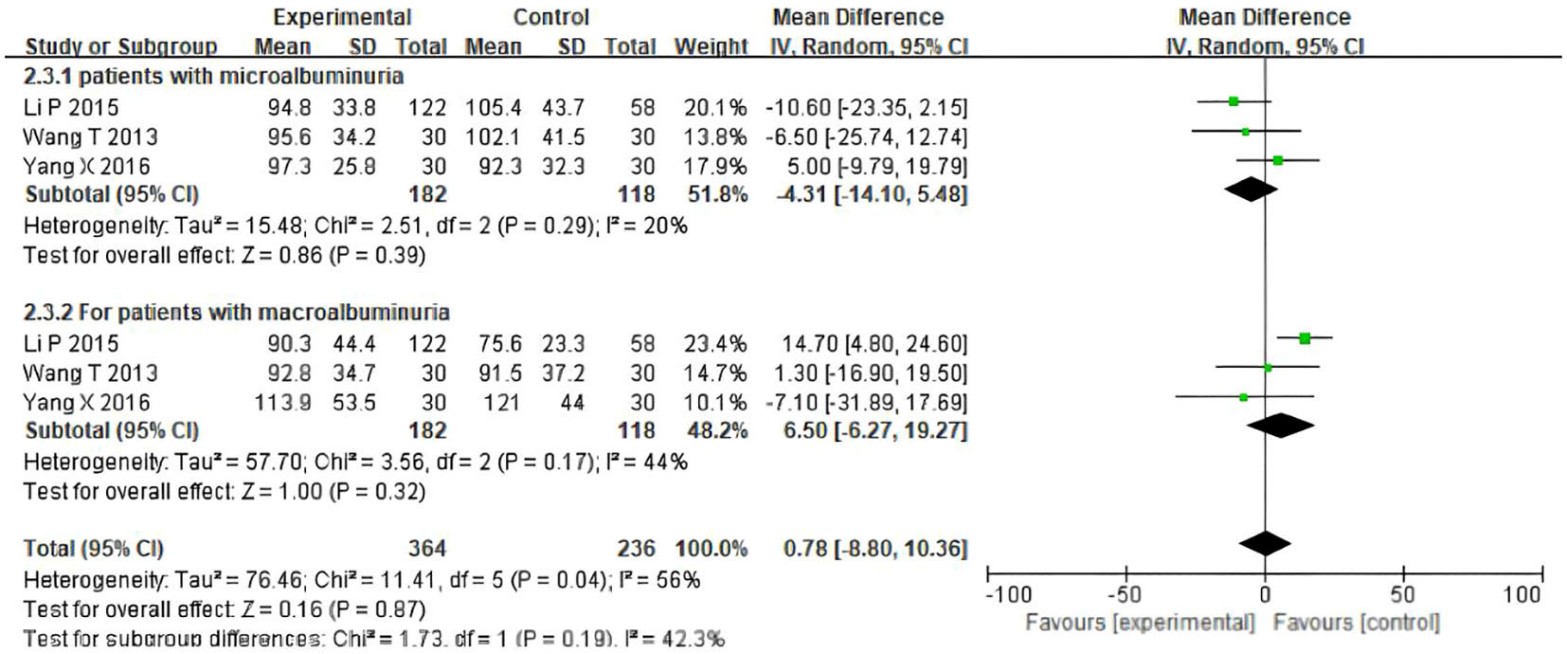
Comparisons of the values of eGFR (microalbuminuria, macroalbuminuria) (after 24 weeks).

To reduce the potential heterogeneity among different types of patients, eGFR levels were measured in patients with microalbuminuria and macroalbuminuria. The results showed that for participants with microalbuminuria, the baseline values of UAER were similar between the TSF group and the placebo group, with no statistically significant difference(OR= -4.31, 95%CI (-14.10, 5.48), P=0.29). For participants with macroalbuminuria, the baseline values of Uaer were similar between the TSF group and the placebo group, with no statistically significant difference (OR =6.50, 95%CI (-6.27, 19.27), P=0.17). As shown in **Figure 5**, after 24 weeks of TSF treatment, there was no significant difference between different types of patients and the control group.

### 3.5 Assessment of adverse events

**Figure 6:**
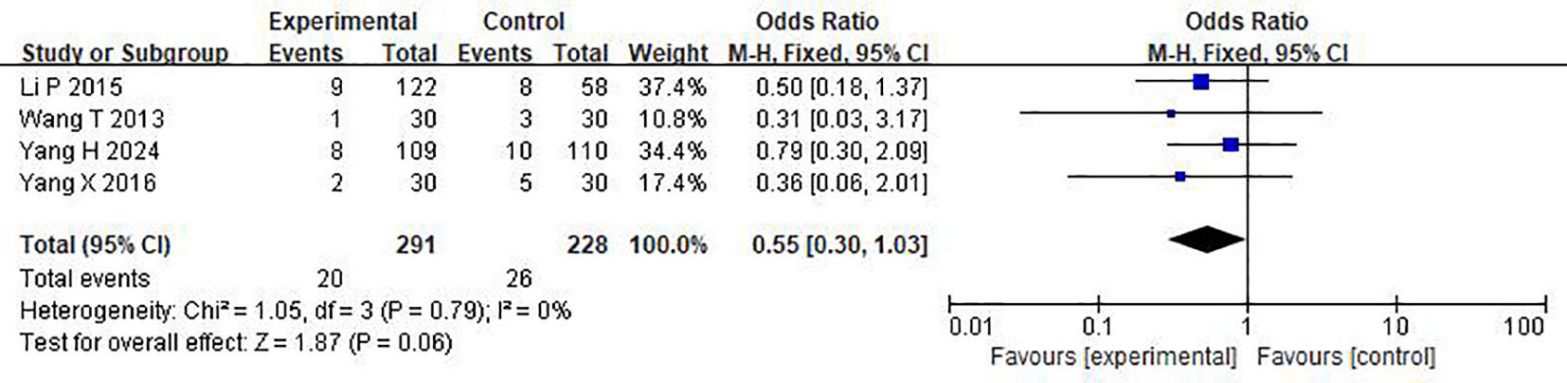
Comparisons of the adverse events (after 24 weeks).

As shown in **Figure 6**, in the treatment of DKD patients with a large amount of proteinuria by TMC compound therapy, the results showed that the probability of adverse experimental occurrence in the intervention group was significantly lower than that in the control group (OR= 0.55(95% CI 0.30-1.03), P=0.79), and there was no significant difference between the two groups. Due to the low heterogeneity, a fixed effect model was used to analyze the OR rate.

### 3.6 Publication bias

Publication bias was visually assessed using funnel plots (**Supplementary Figures S1–S3**). The funnel plots were symmetrical for the values of eGFR, TCM system scores and 24 UP (24 weeks).

### 3.7 Sensitivity analysis

A subgroup analysis was conducted to explore the source of heterogeneity for the value of eGFR. As shown in **Figure 5** and **Supplementary Table S1**, results revealed that no significant difference was found between the patient types.

## 4 Discussion

TSF (Tangshen Formula), a type of traditional Chinese medicine, has been clinically applied as an adjuvant therapy for decades (23). Recent studies show that certain Chinese herbs have renoprotective effects, improving the glomerular filtration rate (GFR) and decreasing proteinuria, especially in patients with microalbuminuria (23–28). Although there was a statistical analysis of published clinical trials, the exact therapeutic effects are yet to be systematically evaluated because of small sample sizes and different protocols among various studies. Therefore, in the present analysis, we performed a wide-ranging online search according to strict inclusion and exclusion criteria, to draw a clear and systematic conclusion.

Our meta-analysis included data from four trials (19–22) that included 639 patients with Diabetic Kidney Disease. The oral dose of TSF in all included studies was 8 grams of TSF twice daily. The combined results showed that TSF achieved more beneficial effects than conventional treatment alone. Compared with conventional treatment alone, TSF could significantly improve UAER and 24 UP (P < 0.05). The study also evaluated the incidence of adverse events during TSF treatment and showed that the incidence of adverse events after TSF treatment was significantly lower than that of the control group within 24 weeks. These results indicate that Tangshen Formula can improve the therapeutic effect of diabetic kidney disease patients. However, there are several factors that may affect the analysis of treatment effects. Therefore, we performed a subgroup analysis to determine the effect of different patient types (microalbuminuria, macroalbuminuria) on the outcome measure (eGFR). Subgroup analysis showed that the therapeutic effect of TSF did not appear to be affected by these factors. However, these surveys have limited research and insufficient sample sizes, which can lead to inadequate assessments. Therefore, these results need to be confirmed by new evidence.

The Tangshen Formula (TSF) is a traditional Chinese herbal medicine used to treat diabetic kidney disease (DKD) (29–31). It primarily consists of raw Astragalus membranaceus, Atractylodes macrocephala, Psoralea corylifolia, Salvia miltiorrhiza, Ligusticum chuanxiong, Trichoderma lucidum, Gongying (dandelion), Pterocarpus indicus, Dendrobium, Kudin tea, and other medicinal herbs. By decocting these ingredients in water and consuming the resulting solution, TSF can achieve therapeutic effects such as tonifying the kidneys and promoting blood circulation.

In our previous clinical trials, we found that TSF reduces macro-proteinuria in stage IV DKD patients, increases the estimated glomerular filtration rate (eGFR), and improves dyslipidemia and abdominal circumference. Diabetic patients frequently exhibit abnormal glycolipid metabolism and impaired renal function. Indicators such as serum creatinine (Scr), β2-microglobulin (β2MG), urinary albumin-to-creatinine ratio (UACR), and estimated glomerular filtration rate (eGFR) can effectively reflect the renal function status of patients. From the perspective of traditional Chinese medicine (TCM), the deficiency of both spleen and kidney functions plays a critical role in the pathogenesis of diabetic kidney disease (DKD). In the Sugar Kidney Formula, raw Astragalus membranaceus is used for its properties of consolidating the exterior to stop sweating, supporting detoxification and promoting tissue regeneration, as well as tonifying qi and uplifting Yang. Atractylodes macrocephala serves to strengthen the spleen and benefit qi, while also drying dampness and promoting diuresis. Psoralea corylifolia is employed for its ability to warm the kidneys, assist Yang, consolidate essence, and reduce urinary frequency. Salvia miltiorrhiza promotes blood circulation, removes blood stasis, unblocks meridians, and alleviates pain.

*In vivo* and *in vitro* experiments have demonstrated that TSF exerts renal protective effects by reducing inflammation and fibrosis, regulating cholesterol metabolism, and promoting autophagy (31–36). By analogy, for each subsequent literature number, add 2 to this basis. In rat and mouse models of DKD, TSF effectively treats renal fibrosis by inhibiting the transforming growth factor beta (TGF-β)/Smad signaling pathway (33). Studies have demonstrated that the potential therapeutic targets of the Sugar Kidney formula for treating renal fibrosis in diabetic nephropathy (DKD) include vascular endothelial growth factor (VEGFA), epidermal growth factor receptor (EGFR), fibronectin 1 (FN1), transforming growth factor beta 1 (TGFB1), signal transducer and activator of transcription 3 (SMAD3), signal transducer and activator of transcription 2 (SMAD2), and others. Additionally, several oxidative stress-related factors have been identified, such as superoxide dismutase 1 (SOD1), heme oxygenase 1 (HMOX1), Kelch-like ECH-associated protein 1 (KEAP1), and nuclear factor erythroid 2-related factor 2 (NFE2L2) (33). NOX proteins are widely distributed throughout the human body, with Nox4 being predominantly expressed in the kidneys. Extensive research has indicated that reactive oxygen species derived from Nox proteins, particularly Nox4, play a critical role in TGF-β-mediated renal interstitial fibrosis. This protein lacks intrinsic catalytic activity and requires the formation of stable complexes with multiple regulatory subunits to exhibit its catalytic function. In unactivated Nox2, the regulatory subunits P40phox, p47phox, and p67phox exist as a complex in the cytoplasm. Upon activation, phosphorylation of p47phox facilitates the translocation of the entire complex to the cell membrane, where it binds to cytochrome b558 (cytb558). Only through the formation of this enzyme complex can catalytic activity be achieved. The TGF-β/Smad signaling pathway is known to be a key pathway in fibrosis development in various organs, including the heart. TGF-β1 has been shown to play a critical pathogenic role in diabetes-associated myocardial fibrosis by activating Smads-dependent signals in diabetic mice, leading to pathological fibrosis (32–38).

TGF-β1 binds to its receptor and activates downstream mediators, including Smad2 and Smad3, to exert biological effects, and is negatively regulated by Smad7 expression (36). Overexpression of TGF-β1 leads to excessive production of extracellular matrix (ECM) proteins and inhibits their degradation, resulting in fibrosis. Additionally, TGF-β1 cooperates with the Wnt protein signaling pathway to control biological activities in various cells (37). The canonical β-catenin-dependent pathway of the Wnt signaling pathway is involved in myocardial fibrosis, where β-catenin forms a complex in the nucleus with transcription factors of T-cell factor/lymphoid enhancer factor (TCF/LEF) to stimulate the transcription of Wnt target genes, thereby leading to ECM deposition (39, 40).

TSF is selected for the treatment of DKD due to its traditional Chinese medicine function of replenishing qi and yin and promoting blood circulation. Subsequent studies have shown that TSF treatment reduces urinary albumin excretion rate (UAER) and decreases glomerulosclerotic and interstitial fibrosis indices in rat models of DKD (34, 41). Further research indicated that the therapeutic effects of TSF in DKD may be attributed, at least partially, to its anti-inflammatory action by downregulating tumor necrosis factor alpha and upregulating pro-inflammatory cytokine interleukin-10 expression, as well as its antifibrotic action by inhibiting the expression of transforming growth factor beta 1 (TGF-β1), enhancing the expression of matrix metallopeptidase 9 (MMP-9), and reducing the expression of collagen type IV (42, 43). Another study also found that TSF appears to exert renal protective effects by improving lipid metabolism. Our preliminary randomized controlled trials suggest that TSF treatment may improve eGFR and reduce proteinuria, particularly in patients with macroalbuminuria (43).

Similarly, there are some limitations to our analysis. Firstly, as an important traditional Chinese medicine, TSF is primarily used in China, which may lead to unavoidable regional bias. Secondly, there is significant heterogeneity among different studies, likely due to the limited number of studies, variations in patient demographics such as gender, age, and geographical locations, as well as differences in treatment duration. However, based on the currently available literature, there is insufficient data to conduct further statistical analysis to evaluate correlations. Thirdly, our results may have inherent bias due to unclear methods of randomization, allocation concealment, and blinding in some of the included trials. Finally, there is limited research and sample size in the studies assessing outcome indicators and safety may introduce analytical bias, thereby compromising the robustness of the findings. These limitations may result in inadequate evaluation of the outcome indicators.

## 5 Conclusion

In summary, the findings of this meta-analysis suggest that TSF can provide effective assistance in reducing urinary protein and improving eGFR in DKD patients compared to conventional treatment. This effect holds true for both patients with microalbuminuria and macroalbuminuria. However, the sample size included in this study is relatively small, so further research is needed to confirm these conclusions.

## Data Availability

The original contributions presented in the study are included in the article/**Supplementary Material**. Further inquiries can be directed to the corresponding author.
