# Supplementary material for: Chinese herbal medicine (Tangshen Formula) formula treatment of patients with diabetic kidney disease: a systematic review and meta-analysis
Source: Front Endocrinol (Lausanne). 2025 Jun 16;16:1522759. doi: 10.3389/fendo.2025.1522759 (PMC12206621; doi:10.3389/fendo.2025.1522759)
Supplement: Supplementary file 1 [file Table1.docx]

Supplementary Table 1. Subgroup analyses of UAER,24 h UP and eGFR between the experimental andcontrol group.

| Parameters | Factors at study level | Patients Con/Exp | Analysis method | Heterogeneity | | Odds Ratio | 95% CI | P-value |
| --- | --- | --- | --- | --- | --- | --- | --- | --- |
|  |  |  |  | I^2^(%) | P-value |  |  |  |
| UAER | Study sample size | | | | | | | |
|  | >100 | 353/226 | Fixed | 0 | 0.98 | -17.98 | -39.15--3.14 | 0.001 |
|  | <100 | 30/30 | Fixed | 0 | 0.34 | -14.38 | -33.98--5.18 | 0.015 |
|  | Type of patients | | | | | | | |
|  | microalbuminuria | 182/118 | Fixed | 0 | 0.81 | -15.94 | -30.67--1.22 | 0.03 |
|  | macroalbuminuria | 182/118 | Fixed | 0 | 0.38 | -17.32 | -25.68--2.49 | 0.001 |
| 24h UP | Study sample size | | | | | | | |
|  | >100 | 353/226 | Fixed | 43 | 0.19 | 0.05 | -0.23-0.33 | 0.009 |
|  | <100 | 30/30 |  | 0 | 0.37 | -0.17 | -0.38-0.03 | 0.007 |
|  | Type of patients | | | | | | | |
|  | microalbuminuria | 182/118 |  | 0 | 0.77 | -0.20 | -0.36--0.05 | 0.01 |
|  | macroalbuminuria | 182/118 |  | 0 | 0.58 | -3.6 | -0.68--0.27 | 0.01 |
| eGFR | Study sample size | | | | | | | |
|  | >100 | 353/226 | Fixed | 0 | 0.35 | 0.73 | -11-12.46 | 0.90 |
|  | <100 | 30/30 | Fixed | 20 | - | -10.6 | -23.35-2.15 | 0.31 |
|  | Type of patients | | | | | | | |
|  | microalbuminuria | 182/118 | Fixed | 20 | 0.29 | -4.31 | -14.10-5.48 | 0.39 |
|  | macroalbuminuria | 182/118 | Fixed | 44 | 0.17 | 6.50 | -6.27-19.27 | 0.32 |
