# Supplementary figures and images for: Chinese herbal medicine (Tangshen Formula) formula treatment of patients with diabetic kidney disease: a systematic review and meta-analysis
Source: Front Endocrinol (Lausanne). 2025 Jun 16;16:1522759. doi: 10.3389/fendo.2025.1522759 (PMC12206621; doi:10.3389/fendo.2025.1522759)

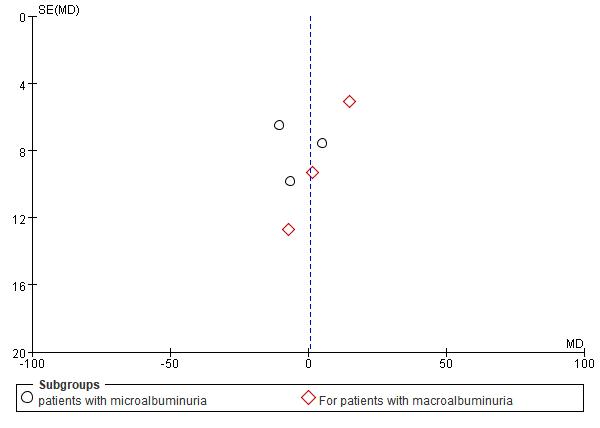

Supplement: Supplementary file 2 [file Image1.jpeg]

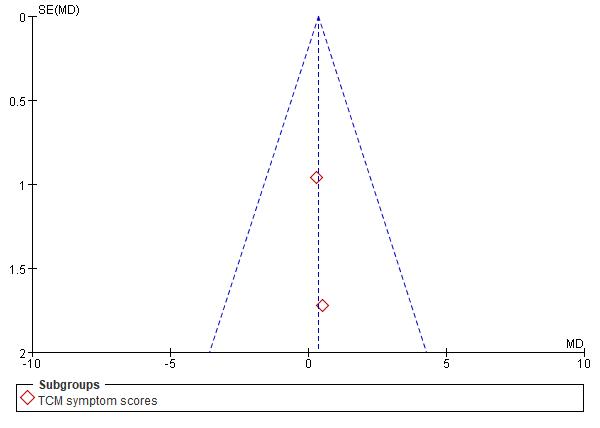

Supplement: Supplementary file 3 [file Image2.jpeg]

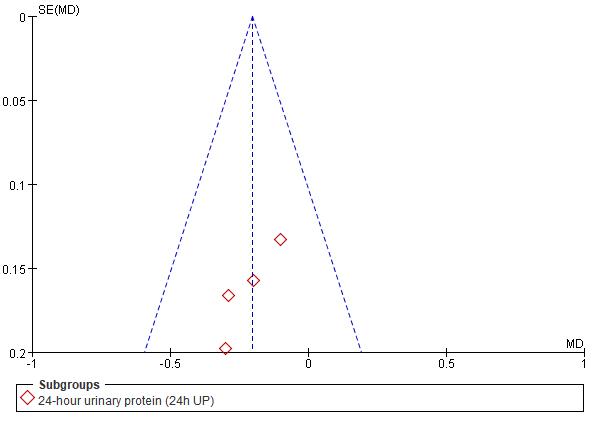

Supplement: Supplementary file 4 [file Image3.jpeg]
